# A comparative study on the comminution behavior of diorite rocks

**DOI:** 10.1016/j.heliyon.2021.e08337

**Published:** 2021-11-09

**Authors:** Gaesenngwe Gaesenngwe, Tirivaviri Mamvura, Gwiranai Danha, Vusumuzi Sibanda

**Affiliations:** a Department of Chemicals, Materials & Metallurgical Engineering, Botswana International University of Science and Technology, Private Bag 6, Palapye 10071, Botswana; b School of Chemical & Metallurgical Engineering, University of the Witwatersrand, Private Bag 3, Wits, 2050, Johannesburg, South Africa

**Keywords:** Diorite, Silicates, Powder x-ray diffraction (PXRD), Crystallography, Aggregate, Construction

## Abstract

In this article factors that affect the comminution behavior of heterogeneous diorite rocks obtained from two quarry locations in Botswana were investigated. Diorite rocks are in great abundance in Botswana and they are increasingly viewed as a relatively inexpensive and reliable alternative construction material to sustain the infrastructure growth in Botswana. The diorite rock samples collected from both the Central and North-Eastern districts were studied for structural similarities and mineral composition. These two are the main factors that influence material hardness, fracture toughness and particle size distribution (PSD) following breakage, which are important material properties in construction applications (Barry & James, 2016). The aim of the investigation was to comminute and compare the behavior of the rock sample types, under similar experimental conditions in a laboratory jaw crusher as well as in a planetary ball mill. The product PSD was used to theoretically determine and compare power requirements. The experimental results show that the diorite rock sample collected from the central region required 41.58 KWht-1 while the one collected from the north-eastern region required 38.33KWht-1 to fragment from a particle feed in the -50 + 40 mm size range to a product in the -11.2 + 6.3 mm size range, which is a typical construction aggregate size range. The diorite sample collected from the central district was largely characterized by amorphous phase constituents and high silicate/quartz content (12.4%) while the north-eastern diorite was characterized by a high crystalline phase percentage and lower silicate/quartz composition (6.94%). The experimental results show that inherent diorite rock properties play a significant role in determining cost of production and product application in the quarry industry of Botswana.

## 1 Introduction

In Southern Africa, Botswana takes pride in having a relatively rapid growing economy characterized by significant domestic investments on infrastructure development projects such as building new road networks, improving old roads, construction of towns and human settlements and water reservoirs like dams. These sectors rely heavily on sands and quarry-based stone products as inputs. The national authority responsible for the environment has recently introduced stringent legislation to monitor compliance of the construction industry in terms of input sourcing. This legislation aims to reduce the reliance of the industry on river sand as it has caused huge ecological and environment damage through erosion and destruction of riverbanks. Diorite rocks have emerged as a viable alternative to river sand. Botswana has an abundant occurrence of diorite rocks that are mainly located in mountainous landscapes as batholiths (McCoy, 2016).

Diorite rock(s) are intrusive rocks in nature, they are classified under Igneous rock which can be plutonic (intrusive) and volcanic (extrusive). Depending on texture of the rock, information about the size, shape and arrangement of interlocking minerals can be deduced from the history of cooling or solidification of their respective magma or lava (McCoy, 2016) and (Carr, 2010). Intermediate composition magma normally consists of 53–65% silica enough to crystallize and form andesite in the diorite complex which typically are considered fine and coarse grained phases. Phaneritic's texture are intermediates of granite (felsic) and gabbo (basaltic) rocks and are largely composed of amphibole, sodium and calcium rich plagioclase feldspar as major or dominant minerals and also in special cases inclusions of pyroxene and biotite in cases where crystallization occurred slowly beneath surface (Jiki and petr, 2019). Therefore, visible minerals are usually large grained with approximately 25–45% dark mineral inclusions contributing largely to material strengthening for fracture toughness and mechanical wear resistance.

The grain structure of diorite when observed under an optical microscope helps in understanding the rock's mechanical breakage patterns (Ramesh, 2018). The particle size of polycrystalline materials as well as the specific material defect density such as voids, dislocation and foreign interstitial components are factors that influence material selection for specific applications. This is because the amount of energy required for breakage is inversely proportional to particle size, grain size and grain orientation of the host rock material (Barry and James, 2016). For various applications such as in streets, highways, railroads ballast, bridges, buildings, sidewalks, sewers, power plants and dams or other parts of the building environment suitable construction material selection is paramount. It is thus imperative that quality-controlled processing of aggregates be undertaken (Rangel et al., 2018). Common problems linked with aggregate quality must be carefully considered such as the constant influence of aggregate particle characteristics on properties of concrete, soundness of aggregate particles in Bituminous mixture, degradation of aggregate leading to the production of deleterious fines, identification of aggregate properties that influence Bituminous coating and adhesion and also the aggregate type and its susceptibility to damage from studded tires (Tutumluer et al., 2018). Other granular materials like crushed stones, sand and gravel are considered among the main source of natural aggregate (Rangel et al., 2018). Sand, gravel and crushed stones nevertheless are mostly used with other binding medium to form concrete, mortar and asphalt or along as in railroad ballast. However, sand and gravel are usually processed based on inherent aggregate size therefore comminution may be omitted depending on the largest gravel or sand particle size specification and the desired product size however, sand may be washed and screened separately to remove dust (Kosmatka & Wilson, 2011).

**Figure 1:**
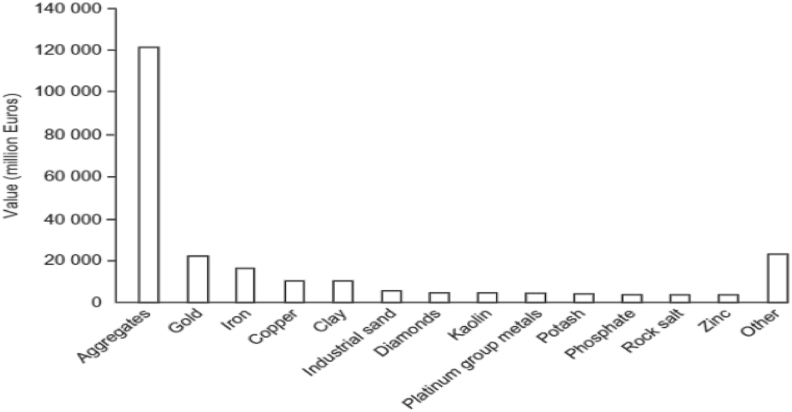
Graph showing the worldwide mineral production from the year 1998.

In the year 1998, the worldwide production of aggregates was approximately 20 billion tons with a total worth of about 120 billion Euros as depicted in Figure 1. Currently the worldwide demand is estimated to be rising by 4.7% annually (Kosmatka & Wilson, 2011).

In this investigation the breakage behavior of diorite rock samples obtained from two separate quarry locations in Botswana is compared. The breakage behavior was also correlated to the mechanical and mineralogical characteristics of the samples.

## 2 Materials and methods

The feed material was collected from Master quarry located in the central part of Botswana and from Panda quarry, located in the northern part of Botswana. Grab sampling method was employed at the quarry sites. Rock samples with an average size of between 40 – 50 mm in diameter were systematically picked from the mining pit. Triangulation plotting was marked out to represent positions of sample collections such that the distance between every sampling point was approximately 5 m and from every collection point, an average mass not exceeding 17 kg was collected. The total canvas sheet mass collection was 50 kg at the pit following drilling and blasting procedures where the material would be excavated and conveyed to the main processing plant for comminution. This sampling method helped in understanding the occurrence of the raw material before controlled laboratory experimentation on selected rocks.

### 2.1 Size reduction method

**Figure 2:**
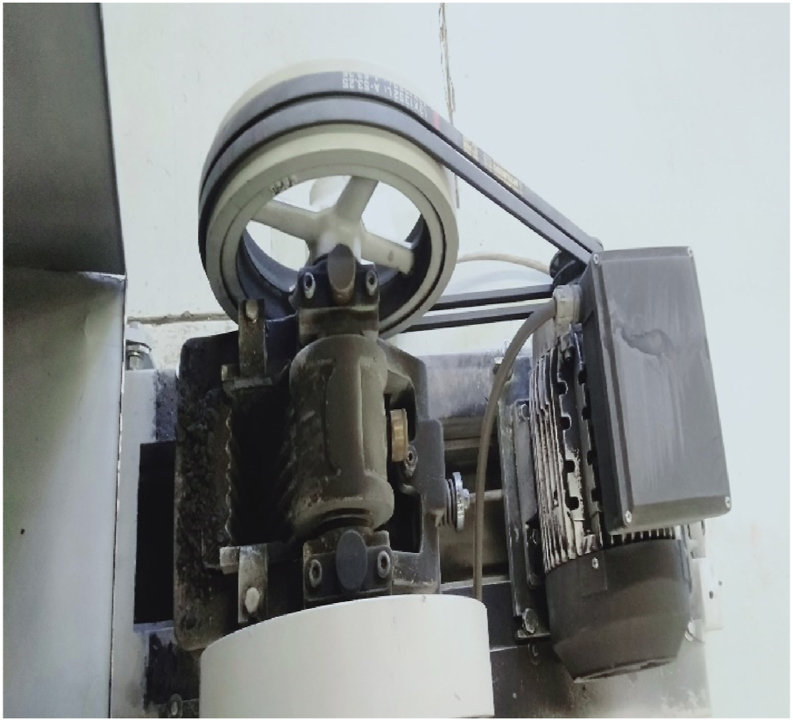
Shows a pictorial laboratory double-toggle Jaw crusher equipment with 100 mm × 150 mm gape opening and 190 depth of crushing chamber.

A laboratory double-toggle Jaw crusher equipment of Figure 2 with 100 mm × 150 mm gape opening and 190 depth of crushing chamber was employed for the primary crushing activities to reduce the size of particles having a total mass of approximately 1400 g. The schematic diagram of Figure 2 is available in (Gupta and Yan, 2006). Following breakdown of the material from the first unit operation, the Jaw crusher, homogenization of the product material was performed using a rifle splitter into 100 g masses hence acquiring 14 batches measured using an electronic balance. Sieve analysis technique was then used to classify and separate the materials into various class ranges according particle size of the fragments. Sieve plates were carefully selected via the squared root of two series method where sieve aperture opening was progressively ranged from -13.6 mm up to +1.7 mm such that the order was in a descending fashion. Auto mechanical sieving was done for each material product batch for 20 min via an electric sieve shaker and mass reporting at each sieve class interval was cumulatively measured and recorded.

**Figure 3:**
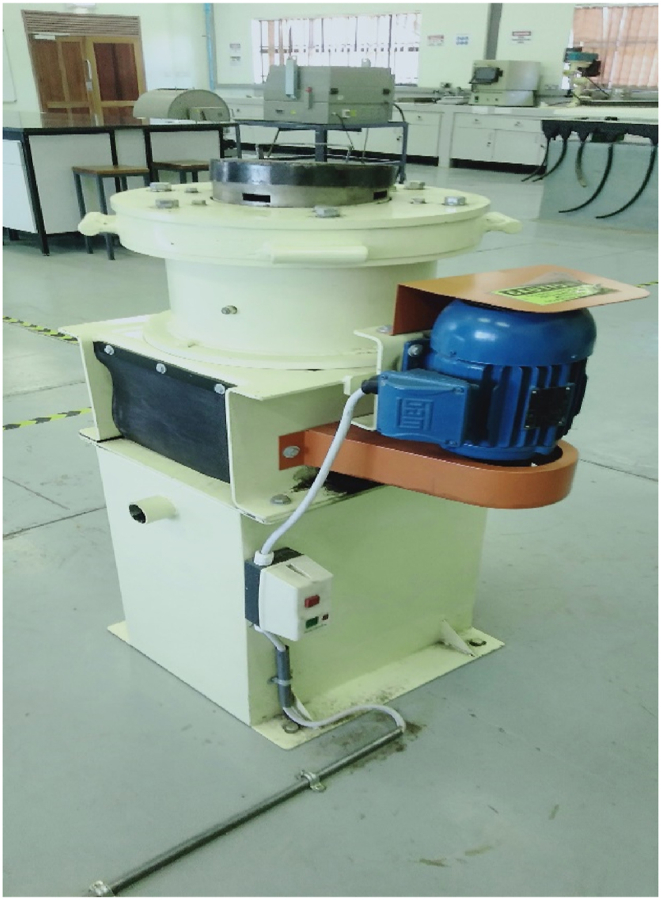
Showing the pictorial image of the laboratory gyratory crusher used for secondary crushing.

The schematic diagram of Figure 3 is provided in (Wills and Napier-Munn, 2006). Primary crusher products were then collectively weighed and recorded then used as feed material for the secondary crushing unit operation. The fragmentation operation was done via a small long shaft laboratory gyratory device having size range 63.5–711 mm, set range 25.4–44.5 mm, rotational speed (rev/min) at 700 and finally power draw of about 2.2 kW as can be seen on Figure 3. Again, using an average sieve aperture size arrangement of -11.2 mm to +6.3 mm, careful selection of the sieve stack was carried out as previously done. The size distribution analysis characteristics for secondary crusher products were then assessed via sieve information of various size classes with the aid of an electronic bathroom scale for accurate measurements.

**Figure 4:**
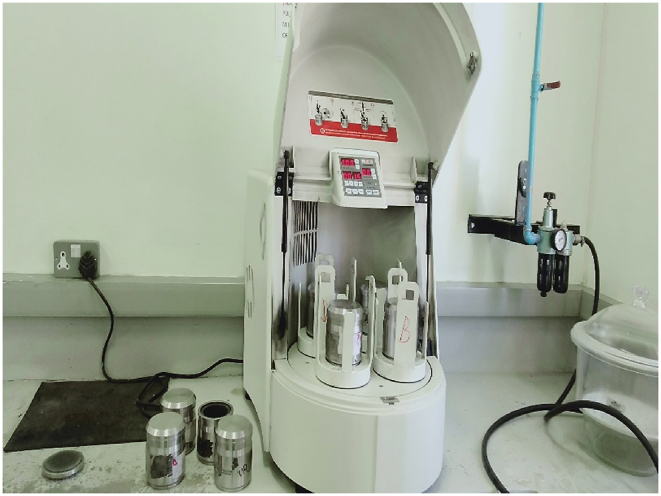
Showing a planetary ball mill for grinding and pulverizing samples depending on device settings. Picture was taken during laboratory experimentation.

Figure 4 shows a planetary ball mill that was set at speed of 250 rpm for 10 min for grinding materials for four cylinders containing the master quarry diorite and panda quarry diorite. The tertiary crushing operation was altogether paramount unit procedure to further fragment the hard igneous material to size below -4.75 mm thus to investigate the effects of impact, attrition and abrasion forces during mill rotation for aggregates in the class size range below -4.75 mm (Gupta and Yan, 2006). Therefore, products of the gyratory crusher were then sifted using a sieve stack arrangement from below -6.3 mm and +0.10 mm after homogenizing the material into 100g samples then cumulatively recording the mass collected at every class interval. Cone and quartering method was adopted when obtaining from the batch material product samples from the secondary crushing unit operation to the next size reduction operation until the tertiary crushing stage. After assessing effects of grinding on material structure and particle size dimensions the mill was again loaded with 15 mm ball sizes at a ball percentage load of 40% and was operated at a rotational speed of 75% of the critical speed and for a grinding time of 10 min. After homogenization product material and standard sieving test analysis particle size distribution behavior of fines were observed thus establishing contrast between the master quarry and panda quarry diorite species. Further sampling was done on the cumulative material product from the planetary ball mill for characterization via a PAN-analytical X'Pert PRO x-ray diffractometer device. Mineralogical composition of any ore plays a significant role in the determination of optimal processing techniques and providing important data for evaluating the amount of energy to be utilized during comminution (Ramesh, 2018).

### 2.2 Energy law model

We employed the Bond's Model (Equation 1) to evaluate the magnitude of the energy expended during the size reduction in reducing the size of the feed material to that of the product material.

(1) $$Energy\;draw=10W_{i}\left[\frac{1}{\sqrt{VMD\left(P\right)}}-\;\frac{1}{\sqrt{VMD\left(F\right)}}\right]KWh/t\;W_{i}=23.0KWh/t$$

**Where**: $W_{i}$ is the Bond Work Index and VMD is the Volume Mean Diameter for products (P) and Feed (F) (Barry and James, 2016).

### 2.3 Characterization method

In-situ x-ray diffraction (XRD) technique was employed to qualitatively identify phase presence in the pulverized samples of diorite rocks. Peak deflection patterns were obtained using a PAN-analytical X'Pert PRO x-ray diffractometer equipped with a sealed proportional detector device with a CuK**α** radiation, operating at a voltage of 45KV and current 40mA. A vertically adjustable multi-purpose sample stage was prepared and the pulverized rock specimen placed symmetrically on an aluminum x-ray sample holder which constantly was aligned to bisect the x-ray beam (Nonaka et al., 2019). Phase analysis of the material was carried out at normal temperature and pressure (20–30 °C) with the Cu K**α** radiation such that, each rock specimen were held under radiation for a period not exceeding 1200s prior a general scan for peak deflection zone identification that was done for 120s in order to guarantee accuracy and stability of the scan to represent the whole rock area under study (Nonaka et al., 2019).

## 3 Results and discussion

### 3.1 Size reduction

Three (3) Sample masses each with mass record of approximately 480g where weighted out via an electric mass balance and cumulatively making a mass of 1400g for the Master and Panda diorite types were carefully measured and used as feed material for the primary crusher. A laboratory jaw crusher with an approximate jaw size of 100 × 150 mm was used and identical crushing conditions were adopted to test the two sample type materials. A comparison of the derivative particle size distribution (PSD) of the Master and Panda diorite ore types crushed using the jaw crusher are as shown in Figure 2.

**Figure 5:**
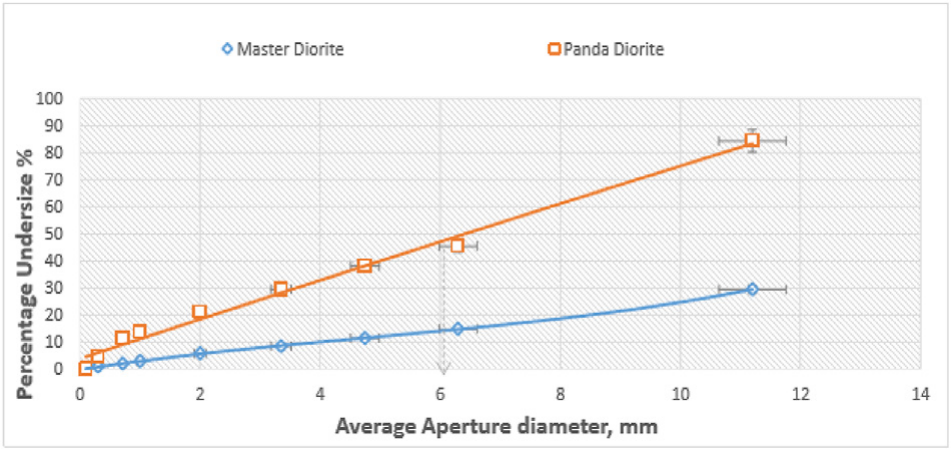
Cumulative % passing vs sieve size for Master and Panda diorite.

Figure 5 shows a plot of cumulative % passing versus sieve size for Master (central quarry) and Panda (north-eastern quarry) product diorite samples from the primary jaw crusher. These results demonstrate that Panda diorite is a much softer material compared to the Master diorite rock. The graph shows that 84.3% of panda diorite material passed a sieve with aperture size 11.2 mm while only 29.4% Master diorite material passed through the same sieve aperture size. Again, 45.5% of Panda diorite material passed a sieve with aperture size of 6.3 mm while only 14.8% of Master diorite material passed through the same sieve aperture size. Choke feeding was avoided because the diorite material is an igneous rock type that is highly dominated by ferromagnesian minerals with high breakage strength therefore pressure on the crusher belt was significantly avoided.

**Figure 6:**
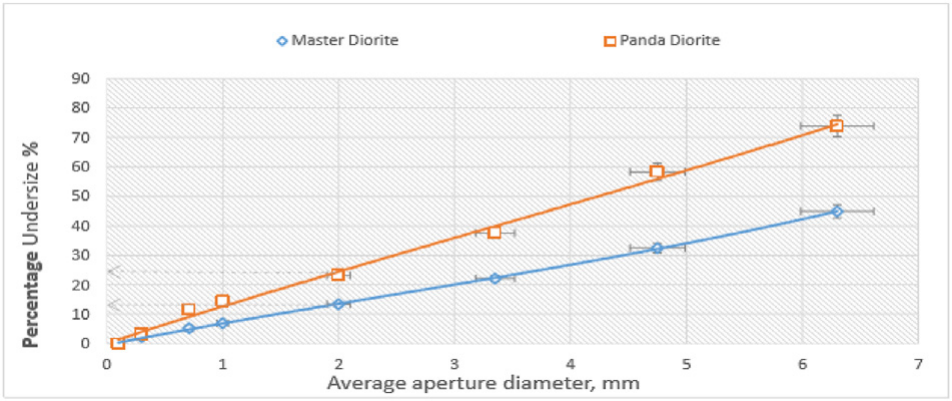
Cumulative % passing vs sieve size for Master and Panda diorite.

Figure 6 shows a comparative cumulative % passing versus sieve aperture size plot for Master (central quarry) and Panda (north-eastern quarry) diorite product samples from the secondary cone crusher. The results reveal that there is an inverse relationship between mechanical strength of material and diorite particle size, as observed by the decrease in the amount of material passing a sieve, with reduction in particle size. Figure 6 is also in agreement with Figure 5 in showing that the Panda diorite is softer compared to the Master diorite. In the secondary cone crusher 58.3% Panda diorite material passed a sieve with aperture size of 4.75 mm while only 32.5% of Master diorite material passed the same sieve size. Also, 23.2% of Panda diorite material passed a sieve with aperture size of 2.0 mm while only 13.4% of Master diorite material passed the same sieve size.

**Figure 7:**
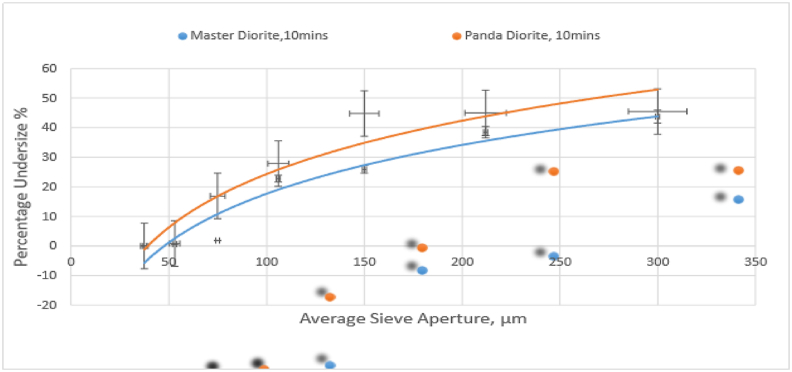
Cumulative % passing vs sieve size for Master and Panda diorite.

Figure 7 shows a plot of cumulative % passing versus sieve aperture size for Master and Panda diorite product samples after milling for 10 min in a ball mill. The results show that 45.0% of Panda diorite material passed through a sieve with aperture size of 212 μm while 38.5% of Master diorite material passed through the same sieve size. Also, 27.8% of Panda diorite material passed through a sieve with aperture size of 106 μm while 22.8% of Master diorite material passed through the same sieve size. The results are consistent in showing that the Panda diorite is softer compared to the Master diorite though the gap between the two materials at milling stage is much smaller than what it was during crushing of larger particles suggesting that the Master diorite becomes progressively easier to crush of softer as the particle size decreases (see Figure 7).

### 3.2 Energy draw

The Bond's energy law model as given in Eq. (1), was used to compare the amount of energy required by the different comminution equipment to effect size reduction of the two diorite rock types from a given feed to a specified product;

#### 3.2.1 Energy drawn by a jaw crusher to reduce size of a master diorite rock

(2) $$Energy=10W_{i}\left[\frac{1}{\sqrt{VMD\;\left(P\right)}}-\;\frac{1}{\sqrt{VMD\;\left(F\right)}}\right]KWh/t\;W_{i}=23.0KWh/t$$

$$Energy=10W\frac{230KWh}{1000\;000g}\left[\left\{\frac{1}{\sqrt{10.99470311}}\right\}-\;\left\{\frac{1}{\sqrt{68.52991527}}\right\}\right]≊\left(0.00023g^{-1}\right)\left[\left(\frac{1}{3.316}\right)-\left(\frac{1}{8.278}\right)\right]KWh/t$$

$$0.00023\left(0.18078592088\right)g^{-1}\approx 0.00004158076\;≅41.58\;Kwh/t\;$$

Energy drawn by a Jaw crusher to reduce size of a Panda diorite rock

(3) $$Energy=10W_{i}\left[\frac{1}{\sqrt{VMD\;\left(P\right)}}-\;\frac{1}{\sqrt{VMD\;\left(F\right)}}\right]KWh/t\;W_{i}=23.0KWh/t$$

$$Energy=10W\frac{230KWh}{1000\;000g}\left[\left\{\frac{1}{\sqrt{15.95}}\right\}-\;\left\{\frac{1}{\sqrt{80.81}}\right\}\right]≊\left(0.00023g^{-1}\right)\left[\left(0.277885007\right)-\left(0.111241656\right)\right]KWh/t$$

$$0.00023\left(0.16664335\right)g^{-1}\approx \;0.000038327\;≅38.33\;Kwh/t$$

#### 3.2.2 Energy drawn by a cone crusher to reduce size of a master diorite rock(s)

(4) $$Energy=10W_{i}\left[\frac{1}{\sqrt{VMD\;\left(P\right)}}-\;\frac{1}{\sqrt{VMD\;\left(F\right)}}\right]KWh/t\;W_{i}=23.0KWh/t$$

$$Energy=10W\frac{230KWh}{1000\;000g}\left[\left\{\frac{1}{\sqrt{5.6416}}\right\}-\;\left\{\frac{1}{\sqrt{10.995}}\right\}\right]≊\left(0.00023g^{-1}\right)\left[\left(0.1194347826\right)-\left(0.111241656\right)\right]$$

$$0.00023\left(0.1194347826\right)g^{-1}\approx \;0.00002746999\;≅27.47\;Kwh/t$$

Energy drawn by a Cone crusher to reduce size of a Panda diorite rock

(5) $$Energy=10W_{i}\left[\frac{1}{\sqrt{VMD\;\left(P\right)}}-\;\frac{1}{\sqrt{VMD\;\left(F\right)}}\right]KWh/t\;W_{i}=23.0KWh/t$$

$$Energy=10W\frac{230KWh}{1000\;000g}\left[\left\{\frac{1}{\sqrt{68.49}}\right\}-\;\left\{\frac{1}{\sqrt{12.95}}\right\}\right]≊\left(0.00023g^{-1}\right)\left[\left(0.1042173913\right)\right]$$

$$0.00023\left(0.1042173913\right)g^{-1}\approx 0.00002396999\;≅23.97\;Kwh/t$$

#### 3.2.3 Energy drawn by a ball mill to reduce size of a master diorite rock

(6) $$Energy=10W_{i}\left[\frac{1}{\sqrt{VMD\;\left(P\right)}}-\;\frac{1}{\sqrt{VMD\;\left(F\right)}}\right]KWh/t\;W_{i}=23.0KWh/t$$

$$\begin{array}{l} Energy=10W\frac{230KWh}{1000\;000g}\left[\left\{\frac{1}{\sqrt{6.0722}}\right\}-\;\left\{\frac{1}{\sqrt{291.6701}}\right\}\right]≊\left(0.00023g^{-1}\right)\left[\left(0.40581394465\right)-\left(0.05855365974\right)\right]\\ 0.00023\left(0.3472602849\right)g^{-1}\approx 0.00007986986\;≅79.87\;Kwh/t \end{array}$$

Energy drawn by a Ball Mill to reduce size of a Panda diorite rock

(7) $$Energy=10W_{i}\left[\frac{1}{\sqrt{VMD\;\left(P\right)}}-\;\frac{1}{\sqrt{VMD\;\left(F\right)}}\right]KWh/t\;W_{i}=23.0KWh/t$$

$$\begin{array}{l} Energy=10W\frac{230KWh}{1000\;000g}\left[\left\{\frac{1}{\sqrt{6.334}}\right\}-\;\left\{\frac{1}{\sqrt{295.9929}}\right\}\right]≊\left(0.00023g^{-1}\right)\left[\left(0.3973387951\right)-\left(0.05812451647\right)\right]\\ 0.00023\left(0.33921427862\right)g^{-1}\approx 0.00007801928\;≅78.02\;Kwh/t \end{array}$$

The energy calculations show that there is a general increase in the size reduction energy requirement as particle sizes get smaller. The energy for Jaw crushing is higher than that of Cone crushing and the most energy is spent during milling. The energy required to crush and/or mill Master (central quarry) material is consistently higher than that required to crush and/or mill Panda (north-eastern quarry) material.

### 3.3 Characterization results

**Figure 8:**
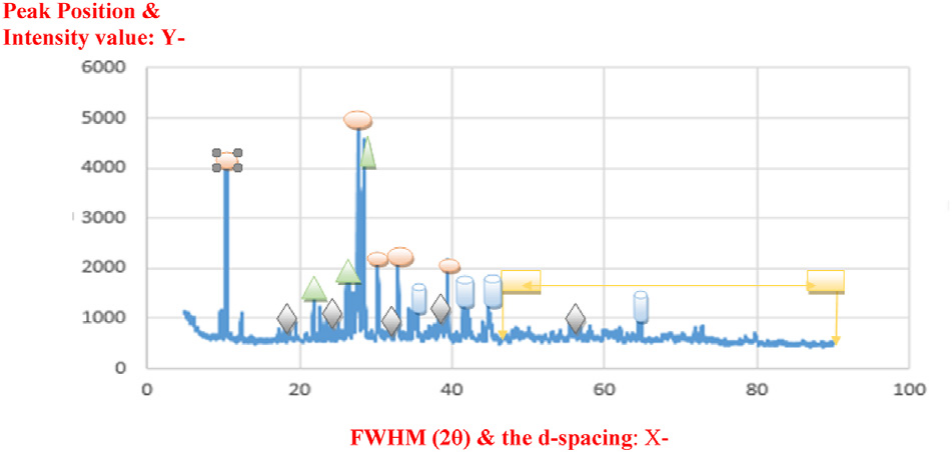
The powder diffraction peak-to-peak information of the master diorite rock samples obtained in Serowe Central Botswana having crystal minerals identified mainly as plagioclase α, berlinite β, bazzite Ω, quartz Φ, lazarite and other trace elements Ψ.

An XRD analysis on the Master (central quarry) diorite samples is shown in Table 1. The results show that crystalline phase composition identified prevalently being plagioclase **α** phase, berlinite **β** complex with a trigonal crystal structure, bazzite **Ω** and quartz **Φ**. Figure 8 shows the peak-to-peak diffraction scan of the Master diorite samples. However, these were only reported peaks that were identified as crystalline phase components which represent a larger section of the rock while an amorphous phase constituent such as clay and other trace constituted the other section.

**Table 1:** Relative percentage abundance of all identifiable crystalline phase components present within the Master diorite rock following an x-ray diffraction scan.

Color	Crystal System	Crystalline Mineral Type	Approximate (%) Abundance & Phases	
Pale Blue	Triclinic	Plagioclase	39	α – Alpha
Brown	Trigonal	Berlinite	23	β– Beta
Grey	Hexagonal	Bazzite	16	Ω - Omega
Yellow	Trigonal-Hexagonal	Quartz	13	Φ- Phi
Light Blue	Cubic	Lazurite	6	Ψ – Psi
Green	Monoclinic-primitive	Chlorite	2	
Dark Blue	Monoclinic	Sericite	1

Powder diffraction for master quarry diorite reveliving peak-to-peak information; the peak position, intensity values, d-spacing value, full width at half maximum FWHM (2θ).

**Figure 9:**
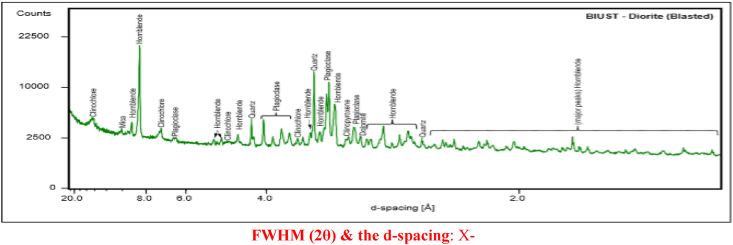
The diffraction graph of the Panda diorite rocks with crystal mineral phases.

The Panda (north-eastern quarry) diorite rock samples when subjected to the same analytical tests and similar results are shown in Table 2 and Figure 9. Table 2 shows that all identifiable crystalline mineral phase components comprise of dark amphibole **α**, plagioclase **β**, clinochlore **Ω**, quartz **Φ**, clinopyroxene **Ψ** and some minor traces of dolomite **θ** and micas **ᴧ**.

**Table 2:** The panda diorite rock mineralogical data and relative percentage abundance of different components.

Color	Crystal System	Crystalline Mineral Type	Approximate (%) Abundance & Phases	
Pale Blue	Monoclinic	Dark amphibole	39	α – Alpha
Brown	Triclinic	Plagioclase	32	β– Beta
Grey	Monoclinic/Triclinic	Clinochlore	12	Ω - Omega
Yellow	Trigonal	Quartz	7	Φ- Phi
Light Blue	Monoclinic	Clinopyroxene	6	Ψ – Psi
Green	Trigonal	Dolomine	3	Θ - Theta
Dark Blue	Monoclinic	Micas	1	Λ- Lamda

Powder diffraction for panda quarry diorite reveliving peak-to-peak information; the peak position, intensity values, d-spacing value, full width at half maximum FWHM (2θ).

### 3.4 Comparative discussion of data from both xrd scanned samples

Figure 9 shows the XRD analysis for the Panda diorite rock, which demonstrates that large mineral crystal grains were identified as dark amphibole **α**, plagioclase **β**, clinopyroxene **Ω** minerals and reporting with a grain size slightly above 1 mm (Nonaka et al., 2019). This phaneritic texture of the rock suggested formation to have occurred at greater depth beneath the surface and rather at slower cooling rates to allow formation of large crystal grains with less clay deposit incorporated within the structure. The comparison of the XRD data analysis for each rock type, show that the Master quarry diorite contained large quantities of plagioclase crystal units (approximately 40%) in contrast with the 32% triclinic plagioclase of Panda diorite. However, when considering the amounts of quartz crystal components, the Master diorite contains 13% of quartz compared to 7% for the panda diorite rocks. High percentage of quartz grains suggests that the rock crystallized at higher temperatures but at rather rapid cooling rates. The Panda diorite rocks showed dark amphibole and plagioclase minerals within its structure as the major constituents, it also has significant clinochlore minerals from the phyllosilicate family of minerals characterized perfect cleavage on their phase orientation known to contribute towards material strengthening. However, according to Hughes (1982) and (Jiki and petr, 2019) the more peak deflection for the Panda rock suggests perfect crystallization hence it exposed valuable mineral crystals within its matrix such as clinopyroxene, dolomite and also traces of mica. The rock crystalized at rather greater depths at high temperatures but at slower cooling rates in comparison to Master Diorite.

## 4 Conclusion

An open comminution circuit was proposed to reduce the size of the diorite rocks from the central and north-east region of Botswana from an average particle size between 40 – 50 mm to a crusher dust material below particle size of -37.5 μm, where cone and quartering sampling method was carried out to analyze specimens for various mineralogical composition via powder x-ray diffraction technology. The size reduction test work was achieved using a laboratory primary jaw crusher, secondary cone crusher and a ball mill. A stack of sieves was used to generate the particle size distribution (PSD) post each size reduction stage. Identical experimental conditions were used in the tests conducted on the two (2) diorite samples under investigation. The central district diorite (Master) demonstrated a higher inherent material strength and a high fracture toughness as interpreted from the cumulative particle size distribution data at all the size reduction stages, which consistently showed that at each screen size there was less material of the Master diorite (from the central region) passing compared to the Panda diorite (from the north-east region). The Jaw crusher required 41.58 *KWh t*−1 of energy to crush Master diorite rocks and 38.33 *KWh t*−1 to breakdown the Panda diorite to the same size while for secondary crushing stage using a gyratory crusher, about 27.47 *KWh t*−1 of energy was required to crush the Master quarry material in comparison to 23.97 *KWh t*−1 of energy required for Panda quarry diorite material. The ball mill required 79.87 *KWh t*−1 of energy to mill the Master diorite rocks and 78.02 *KWh t*−1 to mill the Panda diorite. The mineralogical analysis showed that the Master diorite contained higher amounts of silicates (approximately 13%) compared to Panda diorite (approximately 7 %). Silicate content is an indicator of the difference in the energy required to crush the material. The comminution energy calculations also show that the smaller the particle size the higher the energy required for further size reduction.

## Declarations

### Author contribution statement

Gaesenngwe Gaesenngwe: Conceived and designed the experiments; Performed the experiments; Analyzed and interpreted the data; Wrote the paper.

Tirivaviri Mamvura, Gwiranai Danha & Vusumuzi Sibanda: Analyzed and interpreted the data; Contributed reagents, materials, analysis tools or data.

### Funding statement

This work was supported by the 10.13039/100009145Botswana International University of Science and Technology.

### Data availability statement

The data that has been used is confidential.

### Declaration of interests statement

The authors declare no conflict of interest.

### Additional information

No additional information is available for this paper.

## References

[bib2] Barry W.A., James F.A., Barry w. a. (2016). Mineral Processing Technology:- an Introduction to the Practical Aspects of Ore Treatment and mineral Recovery.

[bib4] Carr J.R. (2010). Andalusite Var Chiastolite Blue wing mountains pershing counly Nevada. Mineral. Rec..

[bib8] Gupta A., Yan D.S. (2006).

[bib9] Hughes C.J. (1982).

[bib11] Jiki K., petr h. (2019). Microstructural and texture formation in commercially pure titanium prepared by crygenic milling and spark plasma sintering. Mater. Char..

[bib22] Kosmatka S.H., Wilson M.L. (2011). Design and Control of Congrete Mixtures: The guide to applications, methods and materials.

[bib14] McCoy L. (2016). The Mineralogy of star trek: the nest series. Mineral. Rec..

[bib15] Nonaka T., Kawaura H., Makimura Y., Nishimura Y. (2019). In situ X-ray Raman scattering spectroscopy of a graphite electrode forlithium-ion batteries. J. Power Sources.

[bib16] Ramesh A.K. (2018).

[bib17] Rangel C., Filho T., Amario R. (2018). Generalized quality control parameter for heterogenous recycled concrete aggregates: a Pilot scale case study. J. Clean. Prod..

[bib19] Tutumluer E., Moaveni M., Qamhia I.A. (2018).

[bib21] Wills B.A., Napier-Munn T.J. (2006).

